# Glutamine Attenuates Inflammation and Stimulates Amniotic Cell Proliferation in Premature Rupture of Membranes-related in vitro Models

**DOI:** 10.1007/s43032-024-01691-9

**Published:** 2024-10-04

**Authors:** Xiang Xiang, Linshen Zhang, Su Li, Yongwei Ren, Daozhen Chen, Lou Liu

**Affiliations:** 1 Department of Toxicology, School of Public Health, Anhui Medical University, Hefei, 230032 China; 2 State Key Laboratory of Reproductive Medicine and Offspring Health, Nanjing Medical University, Nanjing, 211166 China; 3 Research Institute for Reproductive Health and Genetic Diseases, The Affiliated Wuxi Maternity and Child Health Care Hospital of Nanjing Medical University, Wuxi, 214002 China; 4 Affiliated Women’s Hospital of Jiangnan University, Wuxi, 210000 China; 5 Department of Obstetrics, Longgang Maternity and Child Institute of Shantou University Medical College, Shenzhen, 518172 China

**Keywords:** Glutamine, Premature rupture of membrane, Targeted metabolomics, Autophagy, Inflammation, NF-κB, Cell proliferation

## Abstract

Premature rupture of membranes (PROM), with a prevalence of 15.3% in China, frequently results in adverse pregnancy outcomes. In this study, we aimed to identify amino acid metabolites that were differentially expressed in PROM versus healthy controls (HC) using targeted metabolomics and further explored their mechanisms of action with in vitro models.

Inclusion and exclusion criteria were established to recruit 50 PROM and 50 HC cases for targeted metabolomics analysis. Twenty-three amino acid metabolites were quantified in the secretions of the posterior vaginal fornix of pregnant women between 31 and 36 weeks of gestation. Glutamine (0.0216 vs. 0.037 μg/mg, P = 0.003, AUC = 72.1%) was identified as the most differentially expressed amino acid metabolite between PROM and HC groups, and had a negative correlation with the abundance of Gardnerella (r=-0.3868, P = 0.0055), Megasphaera (r=-0.3130, P = 0.0269), and Prevotella (r=-0.2944, P = 0.0380), respectively.

In amniotic epithelial cell and macrophage co-culture model, Glutamine reduced inflammatory cytokines and chemokines expression and suppressed macrophage chemotaxis. In LPS stimulated RAW 264.7 inflammation model, Glutamine inhibited the expression of inflammatory proteins iNOS and COX-2, down-regulated mRNA transcription of TNF, IL-6, and IL-1β, and reduced the production of reactive oxygen species through inhibiting NF-κB signaling pathway, and therefore demonstrated its anti-inflammatory effect. Furthermore, Glutamine protected amniotic epithelial cell from autophagy and stimulated its proliferation, therefore may intensify fetal membrane and prevent PROM in vivo.

Our results suggested that low Glutamine level in vaginal secretion can be used as an indicator for PROM, and local Glutamine supplementation is a potential intervention and prevention strategy for PROM.

**Supplementary Information:**

The online version contains supplementary material available at 10.1007/s43032-024-01691-9.

## Introduction

Premature rupture of membranes (PROM) is defined as the rupture of fetal membranes prior to regular contractions [1]. If PROM occurs before 37 weeks of gestation, it is referred to as preterm premature rupture of membranes (pPROM) [2]. Vaginal microecological imbalance during pregnancy usually results in vaginal inflammation. The membrane tension reflects the pressure of amniotic cavity and the condition of amniotic epithelial cells. Vaginal and intrauterine infection usually increases the pressure in the amniotic cavity and induces apoptosis of the amniotic epithelial cells, and therefore impairs the firmness of the fetal membrane and potentially leads to PROM. When microorganisms travel to upper reproductive tract and infect fetal membranes at cervix, the firmness of membrane is weaken, the risk of membrane rupture increased accordingly, which is one of the leading causes of PROM [3].

The incidence of PROM in China is approximately 15.3% [4]. PROM causes not only neonatal complications [5], but also uterine infections, or even worse systemic infections, sepsis, septicemia, and other severe consequences in pregnant women [6, 7]. In the case of PROM, pathogenic microorganisms may travel up the reproductive tract to the uterine cavity, where severe infections may still occur despite the administration of antibiotics. Therefore, in this study, we were more interested in the population of full-term PROM.

According to our previous 16 S rDNA amplicon sequencing study, the relative abundance of Lactobacillus in the vagina of pregnant women with pPROM reduced, while pathogenic microorganisms, including Gardnerella, Megasphaera, and Prevotella became dominating [8]. This change activated local immune response, and increased the expression of a series of pro-inflammatory cytokines, including IL-1, IL-6 and IL-1β [9]. Feng et al. [10] showed in an animal model that thrombin was detected by immunofluorescence at fetal membrane rupture in cases of vaginal infection with common pathogenic bacteria. There is also evidence of increased thrombin aggregation at the site of fetal membrane rupture. It is believed that certain bacteria can activate thrombin on fetal membranes, leading to the destruction of the intercellular matrix and the rupture of fetal membranes [11]. Additionally, these pathogenic microorganisms interact with the immune barrier of the human vaginal mucosal epithelium, causing abnormal metabolic changes [12]. If the immune barrier cannot effectively resist the invasion of these microorganisms, harmful metabolites increase and beneficial metabolites decrease. For example, Megacoccus is associated with decreased lactose, fructose, and mannitol [13] while Gardnerella and bacterial vaginosis have been associated with elevated short-chain fatty acids, including acetic acid, propionic acid, butyric acid, and succinic acid [14]. Vaginal mucosa isolated from BV patients contains numerous amino acid catabolites. In contrast, *Lactobacillus crispatus* and *Lactobacillus jensenii* dominance of the vaginal microbiota is associated with the presence of intact amino acids and dipeptides, implying that amino acids are used more extensively as a carbon and nitrogen source in BV [12]. During inflammation, cells generate a high level of ROS and consume ATP, which can promote mitochondrial fission and autophagy [15]. Glutamine has been shown to prevent autophagy in various diseases [16–18], however whether it can prevent autophagy in amniotic membrane epithelial cells is not known.

In this study, a prospective cohort was established, and at 31–36 weeks of gestation, secretions from the posterior vaginal fornix of pregnant women were collected. In contrast to the untargeted metabolomics analysis we performed in our previous study [19], we applied targeted metabolomics focusing on 23 amino acids in current study. The purpose of this study was to search for metabolites that can combat inflammation and have a protective effect on PROM, and to screen metabolite markers for early warning of PROM, validate their effects, and develop the guidelines for early intervention and prevention of fetal membranes disruption.

## Materials and Methods

### Study Design and Subject Recruitment

This cohort study was conducted between 2019 and 2021 at the Affiliated Wuxi Maternity and Child Health Care Hospital of Nanjing Medical University. Participants included pregnant women with a single fetus and head presentation who received routine prenatal care and labored vaginally. Preeclampsia, gestational diabetes, malnutrition, multiple gestation, breech presentation, preterm labor experience, short cervical length, preterm labor history, oral or vaginal medication administration within 48 h prior to admission, sexual activity within 48 h prior to admission, and vaginal douching within 48 h prior to obtaining a swab were exclusion criteria for this study.

### Ethical Approval

This study was approved by the Ethics Committee of The Affiliated Wuxi Maternity and Child Health Care Hospital of Nanjing Medical University (2019-02-0402-01) and adhered to the Declaration of China and applicable local regulatory requirements, and the Declaration of Helsinki Principles. It was registered in the Chinese Clinical Trial Registry system (ChiCTR2000034721, registration date: 16 July 2020, https://www.chictr.org.cn/showproj.aspx?proj=56635, accessed on 18 July 2020) and all participants gave written informed consent with their privacy being respected and protected.

### Sample Collection and Processing

All subjects received routine examinations at the Affiliated Wuxi Maternity and Child Health Care Hospital of Nanjing Medical University from the first to the fortieth week of pregnancy. Between weeks 31 and 36, samples were taken from the posterior fornixes, immediately frozen in liquid nitrogen, and stored at -80 °C within five minutes. The calculated prevalence of PROM was 19.05%. A two-sided binomial test was conducted using PASS 11.0 software with a total sample size of 220 (including 42 PROM cases) to determine the minimum sample size required to reach a statistically significant conclusion (α = 0.05, 1-β = 0.8, sensitivity = 72%, specificity = 75%). According to the sensitivity and specificity test results, the actual significance levels achieved were 0.0436 and 0.0427, respectively (the target significance level was 0.05). Then, targeted metabolomics analysis was performed on 50 PROM cases and 50 matched healthy control (HC) cases.

### Targeted Metabolomics

Twenty-three amino acids were chosen for targeted metabolomics. These included Glycine (Gly), L-Alanine (Ala), L-Valine (Val), L-Leucine (Leu), L-Isoleucine (Ile), L-Phenylalanine (Phe), L-Tryptophan (Trp), L-Tyrosine (Try), L-Aspartic(Asp), L-Histidine(His), L-Glutamic (Glu), L-Lysine (Lys), L-Methionine (Met), L-Arginine (Arg), L-Serine (Ser), L-Threonine (Thr), Creatine, L-Proline (Pro), L-Asparagine, (Asn), L-Glutamine, (Gln), 4-Aminobutyric acid (GABA), L-Ornithine hydrochloride (Orn), and Taurine. In brief, the metabolites were extracted from each vaginal cotton swab (adjusted to contain exact 50 mg vaginal secretion proteins), precipitated and mixed with 5 mg/ml amino acid standard for LC-MS analysis. The final results of quantitative analysis for each component were expressed in “μg/mg secretion proteins” (or “μg/mg”, in short).

### Cell Culture

Human amniotic epithelial cells (WISH) and mouse monocyte macrophage (RAW 264.7) were purchased at the National Collection of Authenticated Cell Cultures, China. The high-glucose DMEM culture medium was prepared as following: 88% high-glucose DMEM (Gibco, 10313021), 10% FBS (Gibco, 10099141 C), 1% GlutaMax (Thermo, 350050061), and 1% penicillin-streptomycin (Sigma, 15140-122). Cell culture was kept in incubator with 5% CO_2_ at 37 °C. The RAW 264.7 cell morphology was monitored under a microscope. When the growth state was optimal, the cells were predominantly round and devoid of pseudopods. If more than 10% of the cells showed pseudopods, the M0 phase cells were activated and were, therefore, unsuitable for this portion of the experiment and discarded.

### Transwell Cell Co-Culture and Cytokine Panel Assay

Transwell cell co-culture system (24-well inserts, Corning, 3379) was used to understand the cross-talk between amniotic epithelial cells (WISH) and monocytes/macrophages (freshly isolated from human peripheral blood). About 1 × 10^5^/ml monocytes were seeded in the top insert, while 2 × 10^5^ /ml WISH cells were seeded in the bottom chamber. WISH cell culture medium was set in 4 treatment conditions: no treatment, 10 mM Glutamine treated, 1.5 μg/mL LPS treated, and 1.5 μg/mL LPS plus 10 mM Glutamine treated. The co-culture system was then kept in incubator with 5% CO_2_ at 37 °C for 12 h. After that, the top insert was carefully washed and the membrane was stained with crystal violet to visualize cell migration. The culture medium in the bottom chamber was collected for cytokine panel assay following the manufactory’s instructions (Bio-Rad, 500KCAF0Y). In brief, WISH cell culture supernatant samples were centrifuged at 10,000 rpm for 10 min, and 50 μL of the supernatants were taken for analysis. Incubation of the sample was followed by antibody incubation and detection, color development, and reading on a calibrated Bio-Plex instrument. The detection chip used was 27-plex Bio-Plex Pro Human Cytokine Grp I Panel. The fluorescence values obtained from the standards were used to generate the standard curve and equation under a multi-parameter model. The sample concentration was then calculated using the standard curve and equation.

### Western Blot

1 × 10^6^/mL RAW 264.7 cells were seeded in six-well plates. After growing into monolayers overnight, cells were treated with 2.5 mL of fresh serum-free DMEM medium containing various concentrations of LPS and Glutamine, and whole-cell lysates were collected at various time points. The protein concentration was determined using the BCA kit (Solarbio, China), and the lysates with equal amount of total proteins (30 μg) were loaded for western blotting. The following antibodies were used: iNOS antibody (Abcam, ab283668), COX-2 antibody (Abcam, ab179800), β-actin antibody (Abcam, ab8227), IκBα antibody (CST, 4814 S), Phospho-IκBα antibody (CST, 9246 S), p65 antibody (Abcam, ab32536), PCNA antibody (Abcam, ab92552).

### ROS Fluorescent Probe

RAW 264.7 cells were seeded in 12-well plates at a density of 1 × 10^5^ /mL for ROS fluorescent probe detection. The cells were cultured overnight to form monolayer before 6-hour treatment as described above. After that, the cells were washed twice with PBS before staining. The ROS fluorescent probe (DCFA-CH, Sigma, USA) was diluted to 20 μmol/L, and 400 μL probe was added dropwise to each well with 30 min incubation at 37 ℃ protected from light. The cells were then washed thrice with PBS to remove the fluorescent probe that did not enter the cells. ROS fluorescence intensity of each well was measured using an inverted fluorescence microscope.

### Total RNA Reverse Transcription and Quantitative PCR

RAW 264.7 cells were seeded as described above and treated with LPS and various concentrations of Glutamine for 6 h. Total RNA preparation and reverse-transcription were performed following the manufactory’s standard protocol (Vazyme, China). Quantitative PCR was performed on ABI 7500 (Applied Biosystems) using the following primer sets:

TNF-α: 5′-TTCTCATTCCTGCTTGTGG-3′(F), 5′-ACTTGGTGGTTTGCTACG-3′(R);

IL-6: 5′-GAGGATACCACTCCCAACAGACC-3′(F), 5′-AAGTGCATCATCGTTGTTCATACA-3′(R);

IL-1β: 5′-AGAGCATCCAGCTTCAAAT-3′(F), 5′-CATCTCGGAGCCTGTAGTG-3′(R);

GAPDH: 5′-CCTTCCGTGTTCCTACC-3′(F), 5 ′CAACCTGGTCCTCAGTGTA-3′(R).

### Immunofluorescence (IF)

RAW 264.7 cells were seeded in 24-well plate and treated with LPS and various concentrations of Glutamine for 1 h. The culture medium was aspirated and cells washed with PBS once. To fix the cells, 1 ml of 4% paraformaldehyde was added to each well and incubated for 15 min on ice. After fixation, the cells were washed with washing solution thrice for 3–5 min each time. 1 ml of immunostaining blocking solution and was added and incubated for 1 h at room temperature. NF-κB p65 mouse monoclonal antibody was then added and incubated at 4 °C overnight. The cells were washed thrice for 10 min each time. The anti-mouse Cy3 was added and incubated for 1 h at room temperature, followed by thorough washes, 5-minute cell nuclear staining (DAPI) and further washes. Finally, the slides were sealed and observed under a fluorescence microscope. NF-κB staining was red fluorescence, and the DAPI staining of cell nuclei was blue fluorescence.

### Quantification of Cellular α-KG Level and NADPH/ NADP + Ratio

Cells were seeded into 12-well plates at a density of 10^6^ cells/mL in complete medium overnight and then changed to fresh Glutamine-free medium for 24-hour starving period. After that, cells were treated under various treatment conditions (control, 1.5 μg/mL LPS, or LPS plus 2 mM Glutamine) for 6 hours and then harvested in lysis buffer (∼ 5 × 10^6^ cells in 1 mL lysis buffer) provided by α-KG assay kit (Aidisheng, China, ADS-W-S012) or NADPH/NADP + assay kit (Aidisheng, China, ADS-W-FM015-48). α-KG concentrations (μg/10^4^cell) and NADPH/NADP + ratios were measured following manufactory’s standard protocol.

### Cell Counting Kit-8 (CCK-8) Assay

100μL cells were seeded into 96-well plates at a density of 3 × 10^4^ cells/mL. The medium was changed after 16 h, and LPS and Glutamine of different concentrations were added for stimulation. At 24-hour timepoint, 10μL CCK-8 reagent (Beyotime, China, C0038) was added to each well, and incubated at 37 °C for 30 min. Absorbance at 450 nm was determined using an ELISA microplate reader. The cell proliferation rate was calculated according to the following formula: Cell Proliferation Rate = [(As-Ab)/(Ac-Ab)] × 100%. (As: absorbance of experimental wells, Ac: absorbance of control wells, Ab: absorbance of blank wells).

### Mito-Tracker Green Staining

Mito-Tracker Green (Beyotime, China) was dissolved in anhydrous dimethylsulfoxide (DMSO) to a final concentration of 1mM. Cells were incubated with 200nM Mito-Tracker Green working solution at 37 °C for 15–45 min and then washed with culture medium. Fluorescence microscopy was used to collect images.

### Scanning Electron Microscopy

WISH cells were cultured overnight at 1 × 10^6^/mL during the logarithmic division phase and then changed to Glutamine-free medium for 24 h. They were then treated under 4 conditions for another 24 h: (1) control, (2) 1.5 μg/mL LPS, (3) 1.5 μg/mL LPS + 2mM Glutamine, or (4) 1.5 μg/mL LPS + 10mM Glutamine. The cell pellets were fixed, dehydrated, embedded and sectioned (60–80 nm) for electron microscopy using standard protocol. The sections were double stained with uranium-lead (2% uranyl acetate saturated aqueous solution and lead citrate) for 15 min each and then dried at room temperature overnight. They were examined using a scanning electron microscope, and images were collected for analysis.

### Statistical Analysis

For targeted metabolomics, the results of the quantitative analysis were compared to the median of all amino acid quantification for normalization (the range of normalized data was defined as 0–10), The Wilcoxon test was used to statistically compare the PROM group to the HC group for various between-group differences in the standardized values of the amino acid groups, with *P* < 0.05 indicating a statistically significant difference. ROC (Receiver Operating Characteristic) curve was made in a plot of detection specificity (X-axis) against detection sensitivity (Y-axis), and was used for MS quantitative software optimization. The area under the curve (AUC) for each ROC curve serves as a quantitative metric. The closer of AUC to 100% represented more accurate detection of that amino acid. All experiments were repeated independently three times.

Western blot grayscale values and fluorescence intensity values were quantified using NIH Image J software. qPCR results were exported to Microsoft Excel and the relative mRNA expressions of TNF-α, IL-6, and IL-1β in cells were calculated by 2-ΔΔCT method. One-way analysis of variance (ANOVA) method (GraphPad Prism 9.0) was applied for all statistical analysis of Cytokine panel, α-KG, NADPH/NADP+, CCK-8 assay and Mito-Tracker Green staining results. *P* < 0.05 indicates a statistically significant difference.

## Results

### Demographic and Clinical Data

A total of 50 PROM patients and 50 HC cases were selected to reduce the influence of age and hormone variability. The mothers’ age and gestational week at sampling were matched in a ratio of 1:1. The following clinical characteristics were considered and matched: maternal age, nulliparity, white blood cell count at admission, positive genital cultures at admission, steroid administration, tocolysis treatment, gestational age at sampling, gestational age at delivery, latency from sampling to delivery, BMI in first trimester, BMI in third trimester, baby weight at birth, baby gender, number of deliveries, and spontaneous abortion history. In the study population, neither gestational diabetes nor preeclampsia was observed (Table 1).

**Table 1:** Clinical characteristics of the subjects

Characteristics	Targeted metabolomics (*n* = 100)		
	PROM Cases (*n* = 50)	Controls (*n* = 50)	*P*-Value
Maternal age ^a^	28.86 ± 3.79	29.87 ± 3.81	0.249^†^
Nulliparity	34	47	0.11
White blood cell at admissions (×10^9^/L) ^a^	10.21 ± 3.02	9.89 ± 2.09	0.278^†^
Positive genital cultures at admission	9	11	0.805
Steroid administration	0	0	-
Tocolysis treatment	8	7	0.099^‡^
Gestational age at sampling (weeks)^a^	32.18 ± 1.9	32.65 ± 5.59	0.532^†^
Gestational age at delivery (weeks)^a^	39.64 ± 1.67	39.88 ± 0.95	0.099^†^
Latency from sampling to delivery (weeks) ^a^	5.97 ± 3.36	6.79 ± 0.75	0.126^†^
BMI in first trimester(kg/m^2^)^a^	22.21 ± 3.28	21.35 ± 2.51	0.151^†^
BMI in third trimester(kg/m^2^)^a^ Number of deliveries ^a^	27.55 ± 3.88 1.32 ± 0.49	27.76 ± 2.54 1.57 ± 0.63	0.542† 0.121†
Baby weight at birth (g) ^a^	3056 ± 357.63	3103 ± 376.13	0.170^†^
Baby gender (male/female)	26/24	28/22	0.75^‡^
spontaneous abortion history ^a^	1.09 ± 1.12	0.78 ± 0.89	0.057^†^
gestational diabetes	0	0	-
preeclampsia	0	0	-

PROM: premature rupture of membranes. ^a^ Values are presented as median ± SEM. ^†^ Independent-sample Mann-Whitney U test. ^‡^Fisher’s Exact Test

### Differential Vaginal Metabolites Detected by Targeted Metabolomics

**Fig. 1:**
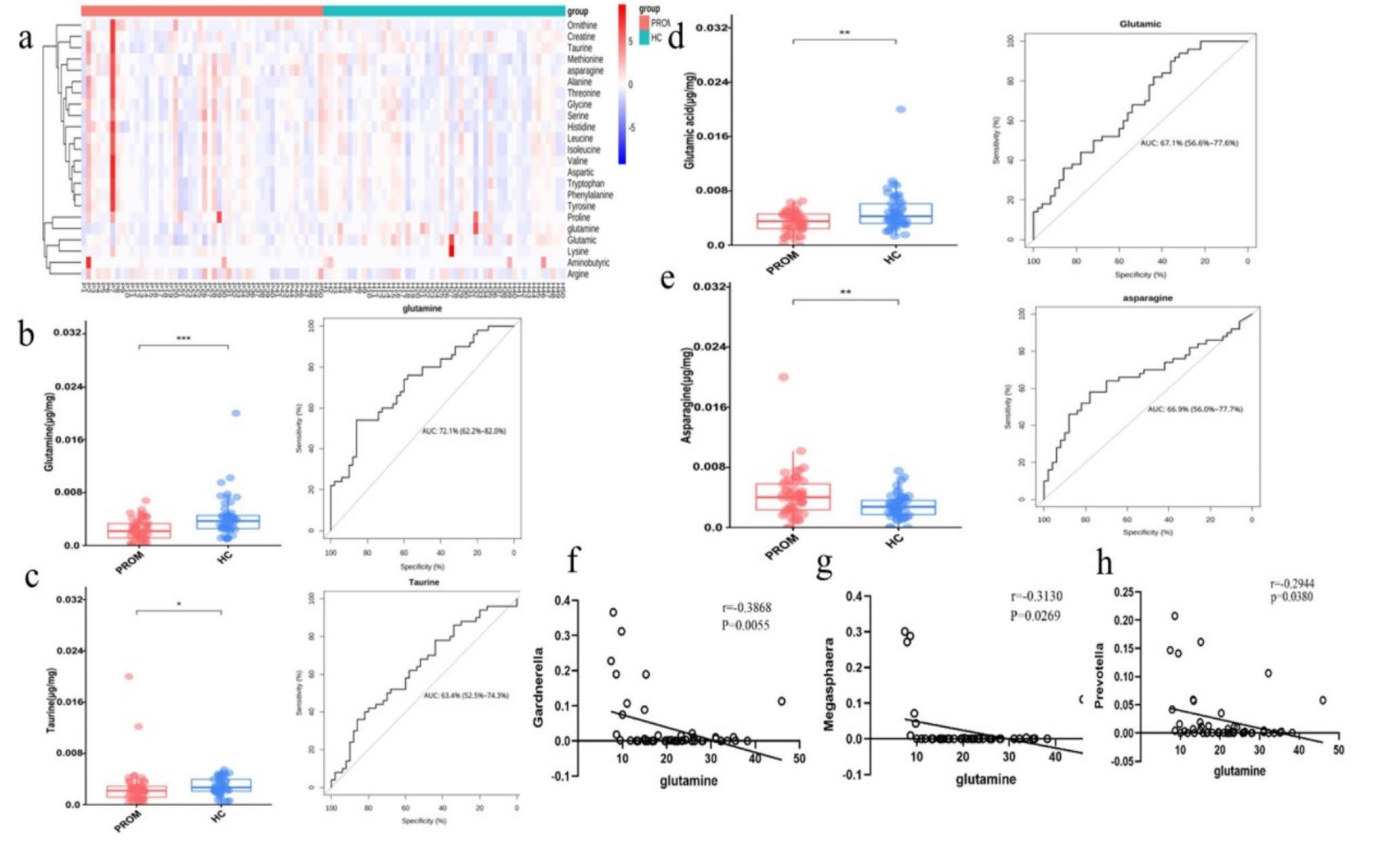
Targeted metabolomics identified differential amino acid metabolites between the PROM group and the HC group. (**a**) Clustered heatmap of 23 amino acids detected by targeted metabolomics. (**b**) Glutamine concentration was significantly lower in the PROM group than in the HC group (1.08 μg/sample vs. 1.85 μg/sample, *P* = 0.003, AUC = 72.1%). (**c**) Taurine concentration was significantly lower in the PROM group than in the HC group (1.09 μg/sample vs. 1.35 μg/sample, *P* = 0.12, AUC = 63.4%). (**d**) Glutamic concentration was significantly lower in the PROM group than in the HC group (1.77 μg/sample vs. 2.13 μg/sample, *P* = 0.03, AUC = 67.1%). (**e**) Asparagine concentration was significantly higher in the PROM group than in the HC group (2.01 μg/sample vs. 1.38 μg/sample, *P* = 0.02, AUC = 66.9%). For AUC values in ROC curves in (**b**-**e**), the closer to 100% indicated better detection. (**f-h**) Glutamine level has a negative correlation with the abundance of Gardnerella(*r*=-0,3838, *P* = 0.0055, **f**), Megasphaera (*r*=-0.3130, *P* = 0.0269, **g**) and Prevotella (*r*=-0.2944, *P* = 0.0380, **h**). *P < 0.5, **P < 0.05

Metabolites concentrations in vaginal secretions were measured quantitatively using targeted metabolomics to determine which metabolites differ between the PROM and HC groups. The targeted metabolomics detection procedure utilized extracted ion chromatogram (XIC) curves as quality control method (Supplemental Figure S1). Each color represented one specific mass-to-charge ratio ion (i.e. one amino acid). The background noise was at e4-e5 range, while signal from all samples and standards was at e6-e7 range, therefore the sample signal was well above background noise which confirmed excellent MS detection. Totally, 23 amino acids were measured by targeted metabolomics which included Glycine (Gly), L-Alanine (Ala), L-Valine (Val), L-Leucine (Leu), L-Isoleucine (Ile), L-Phenylalanine (Phe), L-Tryptophan (Trp), L-Tyrosine (Try), L-Aspartic acid (Asp), L-Histidine (His), L-Glutamic acid (Glu), L-Lysine (Lys), L-Methionine (Met), L-Arginine (Arg), L-Serine (Ser), L-Threonine (Thr), Creatine, L-Proline (Pro), Asparagine (Asn), Glutamine (Gln), 4-Aminobutyric acid (GABA), L-Ornithine hydrochloride (Orn), and Taurine (Fig. 1a). Three amino acid metabolites concentrations were significantly lower in the PROM group than in the HC group: Glutamine (0.0216 μg/mg vs. 0.037 μg/mg, *P* = 0.003, AUC = 72.1%, Fig. 1b), Taurine (0.0218 μg/mg vs. 0.027 μg/mg, *P* = 0.12, AUC = 63.4%, Fig. 1c), and Glutamic acid (0.0354 μg/mg vs. 0.0426 μg/mg, *P* = 0.03, AUC = 67.1%, Fig. 1d). In contrast, Asparagine concentration was significantly higher in the PROM group than in the HC group (0.0402 μg/mg vs. 0.0276 μg/mg, *P* = 0.02, AUC = 66.9%, Fig. 1e).

In our previous 16 S rDNA amplicon sequencing study using the same patient cohort we analyzed vaginal pathogens that might be related to PROM, and we identified pathogens including Gardnerella, Megasphaera, and Prevotella [8]. The abundance of these pathogens had a negative correlation with Glutamine level examined in current study: Gardnerella (*r*=-0.3868, *P* = 0.0055), Megasphaera (*r*=-0.3130, *P* = 0.0269), and Prevotella (*r*=-0.2944, *P* = 0.0380) (Fig. 1f-h). It’s worth mentioning that the levels of Taurine and Glutamic acid showed similar trend although did not reach statistical significance.

### Glutamine Attenuated Macrophage Chemotaxis by Reducing Inflammatory Cytokines and Chemokines Expression in Human Amniotic Epithelial Cells Activated by LPS

**Fig. 2:**
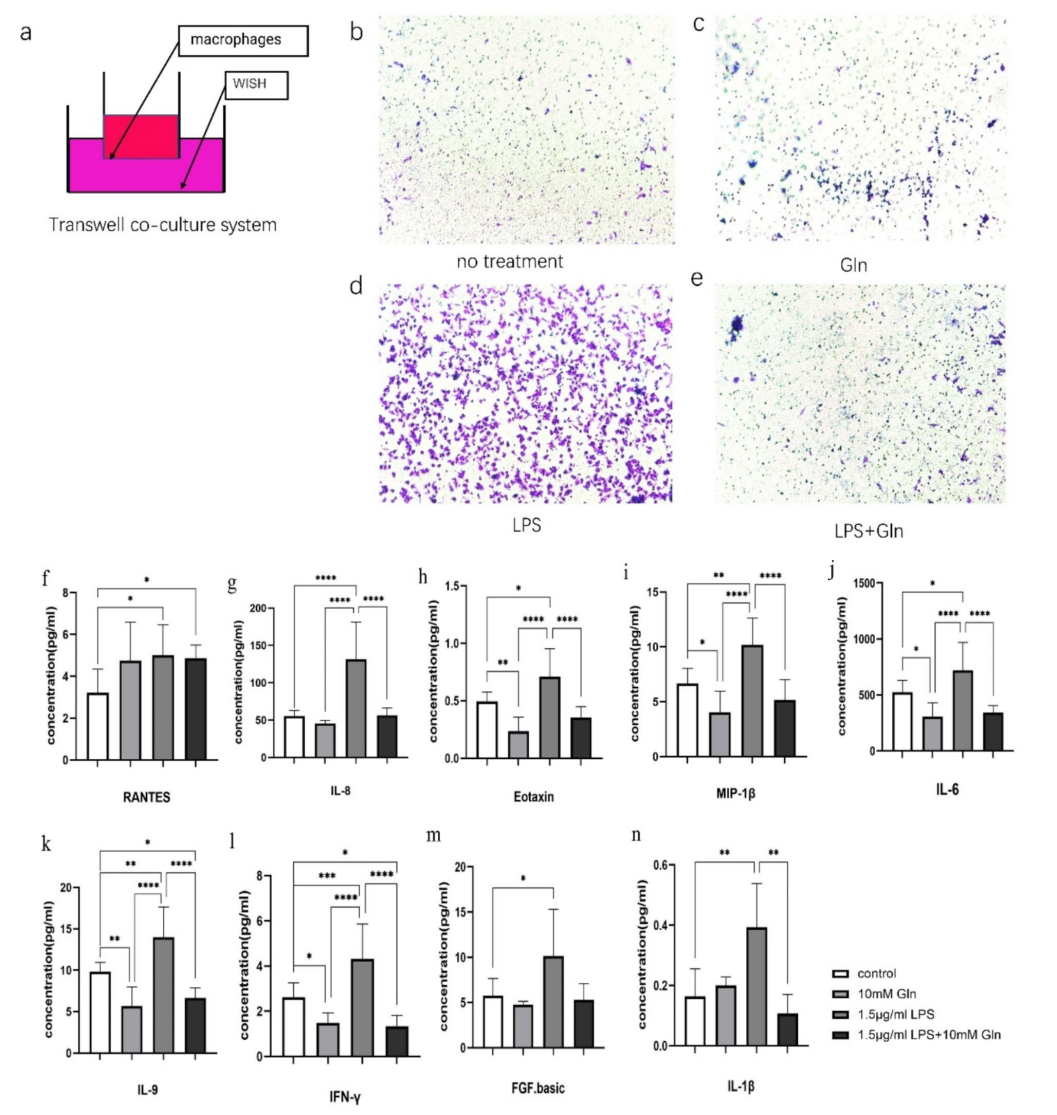
Glutamine attenuated macrophage chemotaxis by reducing inflammatory cytokines and chemokines expression in human amniotic epithelial cells. (**a**) Illustration of the macrophages and amniotic epithelial cells (WISH) transwell co-culture system. (**b-e**) In the co-culture bottom chambers, WISH cells were treated under four conditions: no treatment (**b**), 10 mM Glutamine (**c**), 1.5 μg/ml LPS (**d**), or 1.5 μg/ml LPS + 10mM Gln (**e**). (**f-n**) LPS treatment increased the expression of RANATES (**f**), IL-8 (**g**), Eotaxin (**h**), MIP-1β(**i**), IL-6 (**j**), IL-9 (**k**), IFN-γ(**l**), FGF.basic (**m**), IL-1β (**n**) in the WISH conditioned medium. The upregulation of these cytokine expression was reverted by Glutamine treatment, except for RANTES

Consistent with earlier reports, we observed a large number of macrophage aggregation as the site of rupture of the fetal membranes by immunohistological staining (Data not shown). Based on this, we asked the question whether high level of Glutamine in vagina may suppress inflammation by attenuating macrophage chemotaxis in a simplified in vitro transwell co-culture model (Fig. 2a). In the bottom chamber, human amniotic epithelial cells (WISH) were treated under four different conditions: untreated, 10 mM Glutamine treated, 1.5 μg/mL LPS treated, and 1.5 μg/mL LPS plus 10 mM Glutamine treated. As expected, LPS treatment significantly increased macrophage migration through the membrane, while 10 mM Glutamine successfully attenuated this process (Fig. 2b-e). To understand the mechanism, we then applied Luminex liquid microarray to detect 27 inflammatory cytokines and chemokines in the human amniotic epithelial cell (WISH) culture medium treated with LPS and/or Glutamine. The detection targets included Eotaxin, FGF.basic, G-CSF, GM-CS, IFN-γ, IL-1 β, IL-1α, IL-2, IL-4, IL-5, IL-6, IL-7, IL-8, IL-9, IL-10, IL-12, IL-13, IL-15, IL-17, IP-10, MCP-1, MIP-1α, MIP-1β, PDGF-bb, RANTES, TNF-α and VEGF (see raw data in Supplemental Figure S2). Interestingly, we found 9 cytokines were robustly induced by LPS treatment, and among them, 8 factors were effectively reduced by Glutamine treatment (Fig. 2f-n). Upon bacterial infection, which was mimicked by LPS stimulation, amniotic epithelial cells produced more inflammatory factors and chemokines to attract macrophage chemotaxis to the infection site, while supplement of Glutamine might efficiently attenuate this process.

### Glutamine Protected Macrophages Activation from Inflammation

**Fig. 3:**
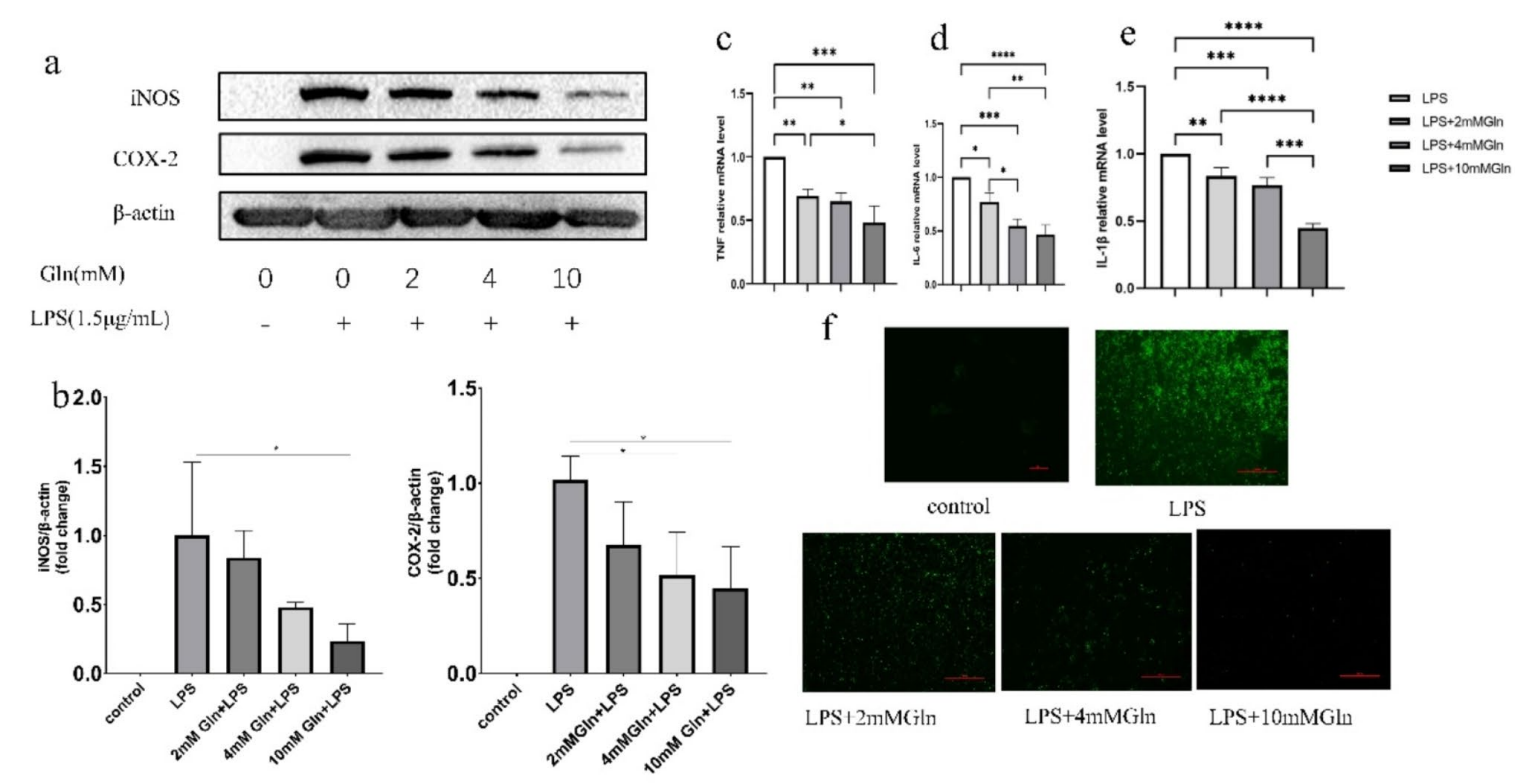
Glutamine reduced inflammation response in macrophages. (**a-b**) Glutamine 6-hour co-treatment reduced inflammatory proteins (iNOS and COX-2) expression in RAW 267.4 cells in a dose-dependent manner. Western bands quantification was shown in **b**. (**c-e**) Glutamine 6-hour co-treatment reduced inflammatory cytokines mRNA transcription in RAW 267.4 cells in a dose-dependent manner. **c**, TNF; **d**, IL-6; **e**, IL-1β. (**f**) Glutamine 6-hour co-treatment reduced intracellular ROS production in a dose-dependent manner. Scale bar, 100 μm. *P < 0.5, **P < 0.05, ***P < 0.01, ****P < 0.001

According to the observation that Glutamine significantly reduced inflammatory factors and chemokines secretion from LPS stimulated human amniotic epithelial cells, we next asked the question whether Glutamine might also protect macrophage from activation upon inflammation. Therefore we established an inflammatory cell model using 1.5 μg/mL LPS to stimulate RAW 264.7 cells and examined whether co-treatment of Glutamine could attenuate the inflammation response. In a 6-hour co-treatment experiment, Glutamine robustly suppressed the expression of inflammatory factors and cytokines in a dose-dependent manner, as examined by either western blot (iNOS and COX-2; Fig. 3a, b) or RT-PCR (TNF, IL-6 and IL-1β; Fig. 3c-e). Moreover, the generation of intracellular ROS is a typical biomarker of M1-type macrophages, and consistently, the Glutamine co-treatment also significantly reduced the production of intracellular ROS examined by fluorescent probe DCFA-CH (Fig. 3f).

### Glutamine Suppressed Inflammation by Inhibiting NF-κB Signaling Pathway Activation

**Fig. 4:**
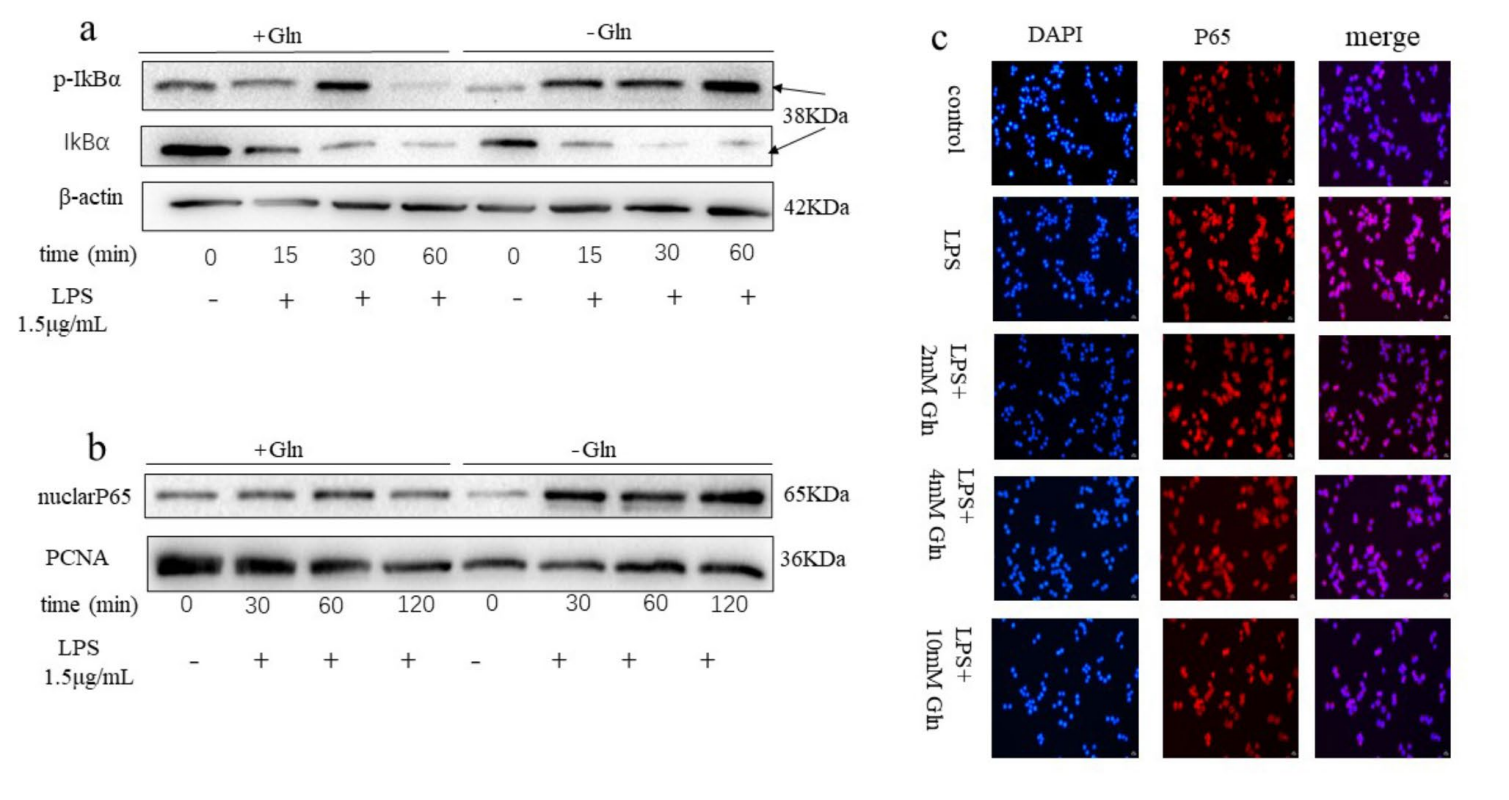
Glutamine inhibited activation of the NF-κB signaling pathway. (**a**) RAW 267.4 cells were treated with 1.5 μg/ml LPS in the presence or absence of 10 μM Glutamine for 30 min. Total and phos-IκBα were examined by western blot. (**b**) RAW 267.4 cells were treated with 1.5 μg/ml LPS in the presence or absence of 10 μM Glutamine for 60 min. Nuclear p65 was examined by western blot. (**c**) RAW 267.4 cells were treated with 1.5 μg/ml LPS in the presence or absence of Glutamine for 60 min. Nuclear localization of p65 was detected by immunofluorescence staining (red). Nucleus was counter-stained with DAPI (blue)

To further explore the underlying mechanism how Glutamine suppressed inflammatory response to LPS stimulation in RAW 264.7 cells, western blot and immunofluorescence experiments were performed to examine the activation of NF-kB signaling pathway, the key regulator of inflammation. The cells were incubated with 1.5 μg/ml LPS in the presence or absence of 10 mM Glutamine co-treatment at 0, 15-, 30-, 60-, and 120-minute time-points. The results showed that the phosphorylation and degradation of the IκBα subunit peaked at 30-minute (Fig. 4a). In contrast, the nuclear translocation of the p65 subunit peaked at 60-minute (Fig. 4b). Interestingly in both experiments, co-treatment of Glutamine attenuated the activation of NF-kB signaling pathway. Consistently, the immunofluorescence staining of p65 subunit clearly showed the activation of NF-kB signaling pathway by LPS stimulation at 60-minute, which could again robustly suppressed by Glutamine co-treatment in a dose-dependent manner (Fig. 4c).

### Glutamine Induced Amniotic Epithelial Cell Proliferation and Prevented it from Autophagy

**Fig. 5:**
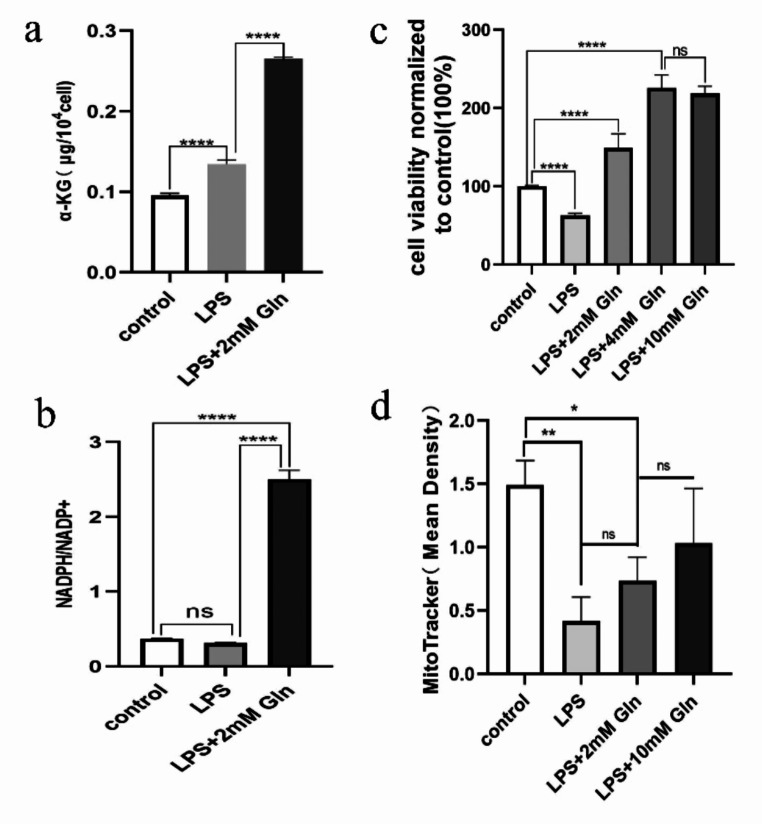
Glutamine fueled cell metabolism and stimulated amniotic epithelial cell proliferation. (**a**) Addition of 2 mM Glutamine significantly increased α-KG level in amniotic epithelial cells. (**b**) Addition of 2 mM Glutamine significantly increased NADPH/ NADP + ratio in amniotic epithelial cells. (**c**) Supplement of various concentrations of Glutamine stimulated amniotic epithelial cells proliferation in a dose-dependent manner within 24 h. (**d**) LPS stimulation significantly reduced mitochondria content in amniotic epithelial cells, while supplement of Glutamine largely rescued the reduction. See original staining in Supplemental Figure S2. *ns*, no statistical difference, *P < 0.5, **P < 0.05, ****P < 0.001

**Fig. 6:**
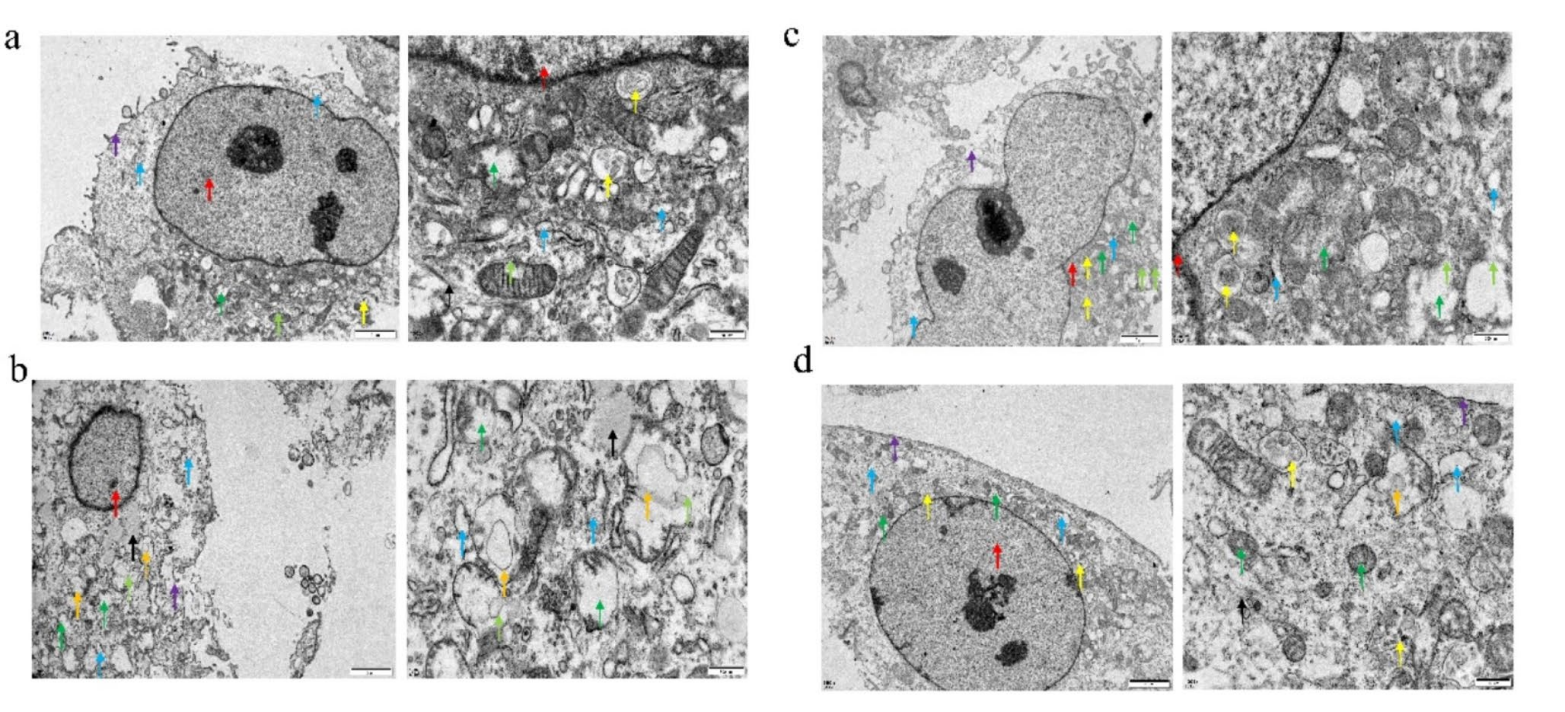
Glutamine protected amniotic epithelial cells from LPS-induced autophagy. (**a**) Amniotic epithelial cells without any treatment. The cell membrane structure was intact, the nucleus shape was regular, the outer and inner nuclear membrane structure was clear, the perinuclear space was normal, and the chromatin in the nucleus was not condensed. There were plenty of mitochondria in good shape. Very few mitochondria had unclear membrane structure, with outer membrane ruptured (light green arrow). (**b**) Amniotic epithelial cells stimulated with 1.5 μg/ml LPS. The cell showed irregular shape, with unclear/ruptured cell membrane structure, so that cytoplasm was exposed. The nucleus was in overall good shape. Massive mitochondria were swollen, and mitochondria cristae were reduced or completely disappeared. The electron density of the matrix was reduced and vacuolated. Some mitochondrial membrane was unclear, with ruptured outer membrane (light green arrow). Some mitochondria underwent mitophagy (orange arrow). (**c**) Amniotic epithelial cells stimulated with LPS and co-treated with 2 mM Glutamine. The cell shape was irregular, and the membrane structure was unclear with some ruptured area, so that cytoplasm was exposed. The nucleus was irregular, and surface was not smooth. The outer and inner nuclear membrane structure was clear, and the perinuclear space was normal with no condensed chromatin structure. Some mitochondria were swollen, mitochondria cristae were reduced, and the electron density of the matrix decreased. A few mitochondria had unclear membrane structures, and the outer membrane was ruptured. A few autophagic lysosomes were found. (**d**) Amniotic epithelial cells stimulated with LPS and co-treated with 10 mM Glutamine. The cell membrane structure was intact, and the nucleus was in good shape. The perinuclear space was normal, and the chromatin was not condensed. A few mitochondria were swollen with mitochondrial cristae reduced. Very few mitochondria underwent mitophagy (orange arrow). Purple arrow, cytoplasmic membrane; Red arrow, nucleus; Yellow arrow, autophagic lysosome; Green arrow, mitochondria; Blue arrow, endoplasmic reticulum; Black arrow, microfilament. Scale bar, 20 μm

Amniotic epithelial cell proliferation is known to play an important role in maintaining the mechanical tension of fetal membrane [20]. This is fueled by cellular metabolic reactions and production of ATP. Given the nature that Glutamine is one of the important energy sources of cell metabolism, we asked the question whether supplement of Glutamine might stimulate amniotic epithelial cell proliferation and/or prevent cell from inflammation (and energy deficient)-related autophagy at the site of PROM, promote membrane repair and reduce the incidence of PROM. We therefore tested in vitro the impact of Glutamine supplement on amniotic epithelial cells (WISH). We noticed that LPS treatment reduced mitochondria content (Fig. 5d) and cell viability (Fig. 5c), which may explain the reduction of “NADPH/NADP+” due to overall change of fitness (Fig. 5b). We were not sure why α-KG level was slightly elevated upon the LPS treatment (Fig. 5a), and this might reflect a feedback response to “unfitness”. Nevertheless, supplement of Glutamine significantly increased the a-KG level, as well NADPH/NADP + ratio, mitochondria content and cell viability. These changes suggested that addition of Glutamine boosted energy metabolism and prevented cells from autophagy. As a result, addition of Glutamine efficiently stimulated cell proliferation within 24 h in a dose-dependent manner (Fig. 5c). Interestingly, when amniotic epithelial cells were stimulated with LPS their mitochondria content was significantly reduced (Fig. 5d). Consistent with this, observation of sub-cellular structures using scanning electron microscope, signs of autophagy induction were clear seen, including swollen mitochondria, decreased mitochondrial cristae, and visibility of autophagy lysosomes (Fig. 6b versus **a**). Autophagy induction by LPS was possibly due to either activation of inflammatory response itself, or inflammation resulted deficiency of intracellular energy. As expected, supplement of Glutamine to the amniotic epithelial cells stimulated with LPS largely rescued the reduction of mitochondria content in a dose-dependent manner (Fig. 6d and Supplemental Figure S3). Meanwhile, scanning electron microscope images also clearly showed the protective effect of Glutamine against autophagy, especially that addition of 10 mM Glutamine almost completely reverse the sub-cellular structure changes induced by LPS (Fig. 6c, d). Altogether, in the LPS-stimulated amniotic epithelial cell culture model, supplement of Glutamine led to clear boost of energy metabolism; therefore, it not only protected cells from autophagy but also induced their proliferation, which may eventually benefit the maintenance of fetal membrane at the site of PROM.

## Discussion

Amino acids can regulate multiple life processes of an organism through a variety of metabolic pathways, including the regulation of glucose and lipid metabolism, the maintenance of homeostasis in the body’s internal environment, the cellular expression of genes involved in signaling, immunity, and stress response, and are also associated with several common diseases. Therefore, we focused on amino acid metabolites when applying metabolomics analysis of vaginal secretion samples from pregnant women in this study. Three amino acids were found significantly decreased in the PROM group: Glutamine, Taurine and Glutamic acid. In contrast, Asparagine was found elevated in the PROM group (Fig. 1). Among these differentially expressed amino acids, Glutamine showed the most significant difference between the PROM and HC groups (*P* = 0.003). Glutamine is mainly synthesized by glutamine synthetase from glutamate and NH4 + while consuming ATP, whereas glutaminase re-catabolizes Glutamine to glutamate and NH4+, producing metabolites such as Glutamic acid and Aspartic acid during catabolism [21]. Lack of Glutamine could impair glycolysis pathway as well as oxidative phosphorylation pathway and therefore reduce overall energy supply. In our study, supplement of 2 mM Glutamine significantly boosted α-KG level (Fig. 5a). The α-KG has been reported to promote macrophage polarization towards M2 type macrophage through epigenetic reprogramming which was beneficial for cell proliferation and healing of membrane. In this progress, NF-κB signal pathway was inhibited and the expression of inflammatory cytokines was downregulated. During the healing process, the fetal membrane near the rupture site became thicker than other part under scanning electron microscope [22, 23]. On the other hand, Glutamine showed negative correlation with the abundance of pathogens that could lead to PROM, such as Gardnerella (*r*=-0,3838, *P* = 0.0055, Fig. 1f), Megasphaera (*r*=-0.3130, *P* = 0.0269, Fig. 1g) and Prevotella (*r*=-0.2944, *P* = 0.0380, Fig. 1h). Since Glutamine was well studied for its roles in metabolism as well as inflammation and immune response, we explored its potential role in regulation of vaginal inflammation and PROM in multiple in vitro cellular models.

Mogami et al. [23] reported the presence of a large number of macrophage aggregates close to the rupture of the fetal membranes, by immunofluorescence staining of M1-type macrophage marker NOS2 and M2-type macrophage marker Arg-1, as well as elevated expression of inflammatory factors such as TNF-α, IL-6, and IL-1β. Microarray analysis of human amniotic epithelial cell culture media revealed that these cells could secrete a variety of chemotactic and inflammatory cytokines to attract macrophage chemotaxis when stimulated by LPS (Fig. 2). When macrophages were recruited to the site of inflammation and activated into M1-type by these inflammatory cytokines, they may release disruptive factors such as MMPs to destroy the fetal membranes and induce the development of PROM. In this study, we showed that supplement of Glutamine could attenuate macrophage chemotaxis.

Endotoxins (LPS) are an essential component of the biological function of pathogenic microorganisms and are found on the surface of most Gram-negative bacteria. Gram-negative microorganisms are the most prevalent pathogens in PROM. In the current study, we used LPS to stimulate RAW 264.7 cells to establish an inflammatory cell model. Using this model, we observed a protective effect of Glutamine on inflammation and also investigated its mechanism. We observed that Glutamine attenuated inflammation in a dose-dependent manner (2–10 mM), decreased the expression of inflammatory proteins iNOS and COX-2, and down-regulated the mRNA transcription of inflammatory cytokines IL-1, IL-1β and TNF-α. Furthermore, Glutamine also reduced ROS production from these LPS-activated macrophages (Fig. 3). Consistent with our study, multiple reports have shown that Glutamine may protect the body against localized inflammation. Using ex vivo experiments, Petrus et al. [24] demonstrated that Glutamine decreased the gene and protein expression levels of pro-inflammatory cytokines in adipose tissue. Soares et al. [25] supplemented cancer patients with Glutamine to alleviate skeletal muscle depletion caused by systemic chronic inflammation. In a separate study, Glutamine decreased the levels of the inflammatory cytokines IL-1β, IL-6 and TNF-α in mice with ulcerative nodular inflammation [26].

The NF-κB nuclear transcription factor family member dimers are sequestered in the cytoplasm by IκB proteins and remain inactive at static state. TNF-α, IL-1β, EGF, LPS, UV, and ROS are the common stimuli that can activate NF-κB pathway. Upon various stimulation, the IκB kinase (IKK) complex is activated, resulting in the removal of the IκB proteins via poly-ubiquitination and proteasomal degradation and therefore NF-κB dimers are freed and translocated to the nucleus and activate target gene transcription [27]. The NF-κB signaling pathway plays a vital role in the regulation of acute and chronic inflammation, as well as immune response [28]. In this study, Glutamine significantly inhibited IκB subunit phosphorylation and p65 subunit nuclear translocation as shown in Western blot and immunofluorescence assays (Fig. 4), indicating that Glutamine could attenuate inflammation by inhibiting the activation of the NF-κB signaling pathway.

Macrophages chemotaxis to the vaginal microenvironment may play opposite roles depends on their activation status. M1-type macrophages typically exaggerate inflammation by producing more cytokines and recruiting more immune cells. If the inflammation is not successfully resolved, these inflammatory cells may release matrix metalloproteinases and destroy fetal membranes, resulting in the development of PROM. In contrast, M2-type macrophages may produce polyamines and proline to promote cell proliferation and collagen production, which may help intensify fetal membranes and prevent the development of PROM [20]. Our current data suggested that Glutamine can not only attenuate inflammation response by suppressing NF-κB pathway (Figs. 3 and 4), but also promote amniotic epithelial cell proliferation and protect them from autophagy under certain conditions (Figs. 5 and 6). Therefore our future study will focus on how Glutamine precisely regulates vaginal macrophage and amniotic epithelial cell function in vivo and eventually serves a protective role in the development of PROM. If our hypothesis is fully validated, Glutamine can be used as an early indicator of PROM, and topical Glutamine supplementation may be developed into a novel intervention method to prevent the development of PROM.

### Limitations of the Study

Unbalanced microecology of the vaginal lining can result in PROM. Inflammation not only affects the amniotic epithelial cells but also activates matrix metalloproteinases to decompose collagen in the fetal membrane tissues, which reduces the tension of the fetal membrane tissues and results in PROM [29]. In this study, only in vitro experiments confirmed the protective effect of Glutamine on cellular inflammation, and no in vivo experiments were conducted to confirm the protective effect of Glutamine on intercellular and collagen inflammation.

## Electronic Supplementary Material

Below is the link to the electronic supplementary material.

Supplementary Material 1

## References

[1] Assefa NE, et al. Risk factors of premature rupture of membranes in public hospitals at Mekele City, Tigray, a case control study. BMC Pregnancy Childbirth. 2018;18(1):386. 30268103 10.1186/s12884-018-2016-6 PMC6162906

[2] Skupski D. Preterm premature rupture of membranes (PPROM). J Perinat Med. 2019;47(5):491–2. 31112509 10.1515/jpm-2019-0163

[3] Paramel Jayaprakash T, et al. High diversity and variability in the vaginal microbiome in women following preterm premature rupture of membranes (PPROM): a prospective cohort study. PLoS ONE. 2016;11(11):1–19. 10.1371/journal.pone.0166794 PMC5115810 27861554

[4] Xia H, et al. The clinical management and outcome of term premature rupture of membrane in East China: results from a retrospective multicenter study. Int J Clin Exp Med. 2015;8(4):6212–7. 26131227 PMC4483966

[5] Brown RG, et al. Vaginal dysbiosis increases risk of preterm fetal membrane rupture, neonatal sepsis and is exacerbated by erythromycin. BMC Med. 2018;16(1):1–15. 10.1186/s12916-017-0999-x PMC5782380 29361936

[6] Yeh CC, Chen CY, Wang PH. Infection and preterm birth. J Chin Med Assoc. 2017;80(9):530–1. 28325575 10.1016/j.jcma.2017.02.004

[7] Gafner M, et al. Risk factors and maternal outcomes following preterm premature rupture of membrane in the second trimester of gestation. Arch Gynecol Obstet. 2020;301(5):1207–12. 32274636 10.1007/s00404-020-05533-2

[8] Liu L et al. Characterization of vaginal microbiota in third trimester premature rupture of membranes patients through 16S rDNA sequencing. Pathogens. 2022;11(8). 10.3390/pathogens11080847 PMC9413980 36014968

[9] Anahtar MN, et al. Cervicovaginal bacteria are a major modulator of host inflammatory responses in the female genital tract. Immunity. 2015;42(5):965–76. 25992865 10.1016/j.immuni.2015.04.019 PMC4461369

[10] Feng L, et al. Infection-induced thrombin production: a potential novel mechanism for preterm premature rupture of membranes (PPROM). Am J Obstet Gynecol. 2018;219(1):pe1011–e10112. 10.1016/j.ajog.2018.04.014 PMC6101239 29660299

[11] Uszyński M, Uszyński W. Coagulation and fibrinolysis in amniotic fluid: physiology and observations on amniotic fluid embolism, preterm fetal membrane rupture, and pre-eclampsia. Semin Thromb Hemost. 2011;37(2):165–74. 21370219 10.1055/s-0030-1270345

[12] Pruski P, et al. Assessment of microbiota:host interactions at the vaginal mucosa interface. Methods. 2018;149:74–84. 29705211 10.1016/j.ymeth.2018.04.022

[13] Noecker C, et al. Metabolic model-based integration of microbiome taxonomic and metabolomic profiles elucidates mechanistic links between ecological and metabolic variation. mSystems. 2016;1(1):00013–5. 10.1128/mSystems.00013-15 PMC4883586 27239563

[14] Stafford GP, et al. Spontaneous preterm birth is associated with differential expression of vaginal metabolites by lactobacilli-dominated microflora. Front Physiol. 2017;8:615–29. 28878691 10.3389/fphys.2017.00615 PMC5572350

[15] Jin JY, et al. Drp1-dependent mitochondrial fission in cardiovascular disease. Acta Pharmacol Sin. 2021;42(5):655–64. 32913266 10.1038/s41401-020-00518-y PMC8115655

[16] Thomas K et al. Glutamine prevents acute kidney injury by modulating oxidative stress and apoptosis in tubular epithelial cells. JCI Insight. 2022;7(21). 10.1172/jci.insight.163161 PMC9675453 36107633

[17] Kim MH, Kim H. The roles of glutamine in the intestine and its implication in intestinal diseases. Int J Mol Sci. 2017;18(5). 10.3390/ijms18051051 PMC5454963 28498331

[18] Yin K, et al. Polystyrene microplastics up-regulates liver glutamine and glutamate synthesis and promotes autophagy-dependent ferroptosis and apoptosis in the cerebellum through the liver-brain axis. Environ Pollut. 2022;307:119449. 35550135 10.1016/j.envpol.2022.119449

[19] Liu L et al. Detection of vaginal metabolite changes in premature rupture of membrane patients in third trimester pregnancy: a prospective cohort study. Reprod Sci. 2020;28(2):585–594. 10.1007/s43032-020-00338-9 PMC7537967 33025530

[20] Menon R, Richardson LS. Preterm prelabor rupture of the membranes: a disease of the fetal membranes. Semin Perinatol. 2017;41(7):409–19. 28807394 10.1053/j.semperi.2017.07.012 PMC5659934

[21] Cruzat V, et al. Glutamine: metabolism and immune function, supplementation and clinical translation. Nutrients. 2018;10(11):1–31. 10.3390/nu10111564 PMC6266414 30360490

[22] Liu PS, et al. Alpha-ketoglutarate orchestrates macrophage activation through metabolic and epigenetic reprogramming. Nat Immunol. 2017;18(9):985–94. 28714978 10.1038/ni.3796

[23] Mogami H, et al. Healing of preterm ruptured fetal membranes. Sci Rep. 2017;7(1):1–15. 29030612 10.1038/s41598-017-13296-1 PMC5640674

[24] Petrus P, et al. Glutamine links obesity to inflammation in human white adipose tissue. Cell Metab. 2020;31(2):375–90. 31866443 10.1016/j.cmet.2019.11.019

[25] Soares JDP, et al. Dietary amino acids and immunonutrition supplementation in cancer-induced skeletal muscle mass depletion: a mini-review. Curr Pharm Des. 2020;26(9):970–8. 32067606 10.2174/1381612826666200218100420

[26] de Oliveira Santos R, et al. L-glutamine and physical exercise prevent intestinal inflammation and oxidative stress without improving gastric dysmotility in rats with ulcerative colitis. Inflammation. 2021;44(2):617–32. 33128666 10.1007/s10753-020-01361-3

[27] Xia Y, Shen S, Verma IM. NF-kappaB, an active player in human cancers. Cancer Immunol Res. 2014;2(9):823–30. 25187272 10.1158/2326-6066.CIR-14-0112 PMC4155602

[28] Zhao H, et al. Inflammation and tumor progression: signaling pathways and targeted intervention. Signal Transduct Target Ther. 2021;6(1):1–46. 34248142 10.1038/s41392-021-00658-5 PMC8273155

[29] Litwiniuk M, et al. The MMP-9/TIMP-1 imbalance and the reduced level of TGF-β in the cervical area of amniotic membrane is a possible risk factor of PROM and premature labor - proof-of-concept study. Ginekol Pol. 2017;88(7):379–84. 28819943 10.5603/GP.a2017.0071

