# Supplementary material for: Glutamine Attenuates Inflammation and Stimulates Amniotic Cell Proliferation in Premature Rupture of Membranes-related in vitro Models
Source: Reprod Sci. 2024 Oct 4;32(3):854–66. doi: 10.1007/s43032-024-01691-9 (PMC11870963; doi:10.1007/s43032-024-01691-9)
Supplement: Supplementary file 1 — Supplementary Material 1 [file 43032_2024_1691_MOESM1_ESM.docx]

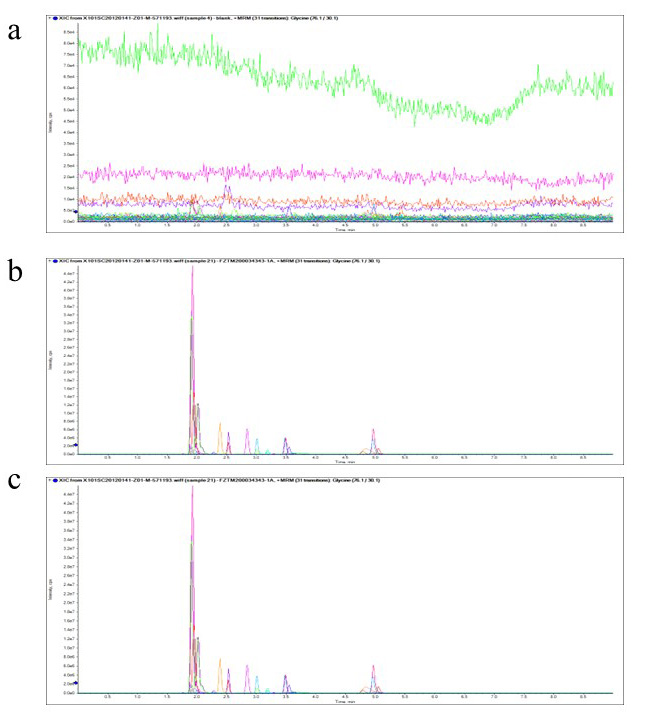


**Figure S1. Original chromatograms for targeted metabolomics.** (**a**) The blank XIC plot of 23 amino acids, represented background noise of detection at e4-e5 range. (**b**) The sample XIC plot signal was at e6-e7 range. (**c**) The standards XIC plot signal was at e6-e7 range. Both were well above background noise which confirmed excellent MS detection.


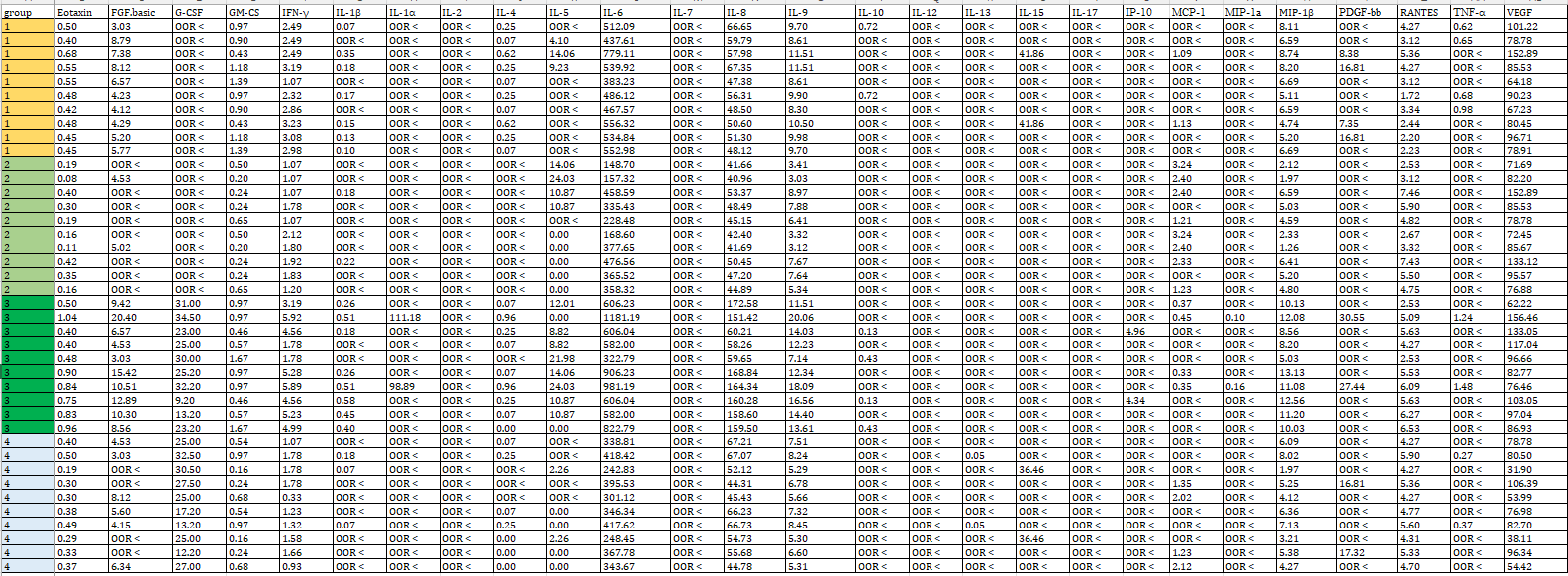


**Figure S2. Raw data of Luminex liquid microarray of 27 inflammatory cytokines.** (**1**) no treatment. (**2**) 10 mM Glutamine treated. (**3**) 1.5μg/ml LPS treated. (4) 1.5 μg/ml LPS plus 10 mM Glutamine treated. The unit of inflammatory cytokines is pg/ml. OOR< indicated the cytokine concentration was below detection range.


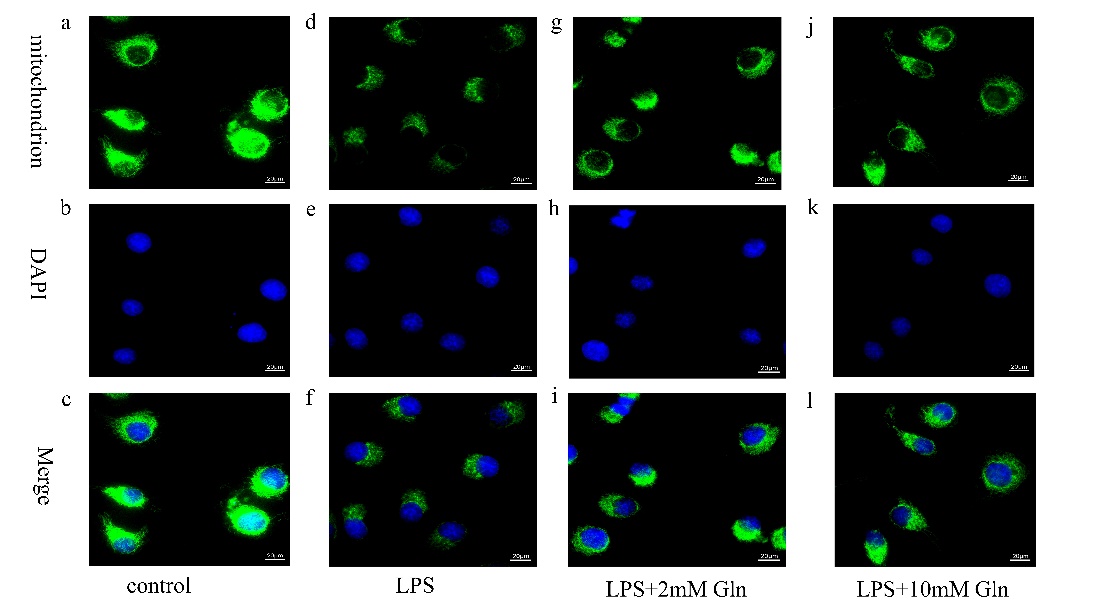


**Figure S3. Original MitoTracker mitochondrial probe staining.** Mitochondrial content (green fluorescence) was quantified and presented in **Figure 5d**. Scale bar, 20 μm.
